# Understanding Dynamic Status Change of Hospital Stay and Cost Accumulation via Combining Continuous and Finitely Jumped Processes

**DOI:** 10.1155/2018/6367243

**Published:** 2018-06-10

**Authors:** Yanqiao Zheng, Xiaobing Zhao, Xiaoqi Zhang

**Affiliations:** ^1^School of Finance, Zhejiang University of Finance and Economics, China; ^2^School of Data Sciences, Zhejiang University of Finance and Economics, China

## Abstract

The Coxian phase-type models and the joint models of longitudinal and event time have been extensively used in the studies of medical outcome data. Coxian phase-type models have the finite-jump property while the joint models usually assume a continuous variation. The gap between continuity and discreteness makes the two models rarely used together. In this paper, a partition-based approach is proposed to jointly model the charge accumulation process and the time to discharge. The key construction of our new approach is a set of partition cells with their boundaries determined by a family of differential equations. Using the cells, our new approach makes it possible to incorporate finite jumps induced by a Coxian phase-type model into the charge accumulation process, therefore taking advantage of both the Coxian phase-type models and joint models. As a benefit, a couple of measures of the “cost” of staying in each medical stage (identified with phases of a Coxian phase-type model) are derived, which cannot be approached without considering the joint models and the Coxian phase-type models together. A two-step procedure is provided to generate consistent estimation of model parameters, which is applied to a subsample drawn from a well-known medical cost database.

## 1. Introduction

Rising expenditures and constraints on health care budgets have prompted the development of a variety of methods for the analyses of hospital charge and length of stay (LOS) as discussed in Gold [1], Lipscomb et al. [2], and Lin et al. [3]. Correctly fitting the charge and LOS data is a critical step in optimizing the allocation of healthcare resources. But due to the protection of private information, the detailed information regarding the treatment process that patient experience in hospital is not available from many well-known medical outcome databases, like the New York State's Statewide Planning and Research Cooperative System. The missing longitudinal information of the treatment process makes it more challenging to generate good fitting; meanwhile it becomes demanding to have a dynamic model, through which effective inference can be made against the hidden treatment process. To that goal, a bunch of stochastic-process-based models have been well developed and applied to analyze the medical datasets.

The continuous-time Phase-Type (PH) model has been widely used in the study of hospital charge and LOS data. Many authors focus in particular on a special subclass of PH model/distribution, namely, the Coxian phase-type (CPH) model/distribution Tang [4]; Faddy et al. [5]; Marshall et al. [6–8]; Fackrell [9]. Unlike other popular theoretical distributions widely used in inpatient data, such as log-normal and gamma distribution, the CPH model/distribution not only provides a theoretical distribution that can be used to fit the empirical data, but also gives us a sketch of the treatment dynamics that patient experience in hospital. In fact, from CPH models, we can track the pathways that patient went through in different medical stages (characterized by the discrete set of phases in the PH model) during a hospital stay. The pathway information makes it possible to cluster patients and facilitate the use of healthcare process improvement technologies, such as Lean Thinking or Six Sigma McClean et al. [10, 11].

The other popular approach to study hospital charge and LOS is through dynamically modelling the charge accumulation process and the determination of the time to discharge, which belongs to a more general class of joint models of the longitudinal measurements and time to event, Ibrahim et al. [12]; Tsiatis and Davidian [13]; Henderson et al. [14]; Kim et al. [15]; Sousa [16]; Lawrence Gould et al. [17]. In medical cost studies, the charge accumulation is a monotonic nondecreasing process; the joint model used in this case is reduced to a class of random growth with random stopping time (RGRST) models.

Like CPH models, the RGRST models do also capture the treatment dynamics that patient experience in hospital. But in contrast to tracking the pathways of patient moving through different medical stages, the RGRST models focus more on describing how patient and/or doctor makes the discharge decision in reaction to the change of actual charge level and the length of time that patient has stayed in hospital. Therefore, the story of RGRST models is more about the behavioural patterns of patient/doctor behind the treatment dynamics, while the story of CPH models is more on the medical side.

It is natural in this paper to think of the possibility of combining CPH models and RGRST models together in order to extract more information regarding the discharge decision-making on different medical stages. However, there is a natural gap between the two models. The CPH model is a finitely jumped stochastic process in essence, while the charge accumulation in the RGRST model is continuous. It is not trivial to combine a jump process with a continuous process. To deal with that difficulty, we propose a partition-based approach with each partition cell determined by solving a boundary differential equation. These boundary differential equations are subtly designed to merge the continuous charge into discrete “phases” involved in a Coxian phase-type model. In sum, the main contributions of this paper are as follows:

(i) We show that there is a natural way to convert a special subclass of RGRST models to CPH models.

(ii) We propose an algorithm to estimate the transition matrix of the CPH model converted from a given RGRST model and the parameters involved in that RGRST model.

(iii) Based on the correspondence between RGRST models and CPH models, we derive a variety of different measures of the “cost” of staying in a medical stage at each time. That “cost” information is important for the purpose of insurance payment and healthcare process improvement.

McClean et al. [11] tried a different way to incorporate the charge accumulation process into a CPH model. But in their work only the case that the charge accumulation process adopts a piece-wise linear form was discussed. It turns out that the piece-wise linear assumption is quite restrictive while crucial to their main result. Without it, the matrix technique in McClean et al. [11] is no longer applicable to achieve the *n*th order moments of total charge for *n* > 1, while our differential-equation-based approach does still work. In fact, we believe our method extends the work of McClean et al. [11] in the following two aspects.

(1) Instead of being piece-wise linear, we consider a much more general situation in which the charge accumulation process can take arbitrary forms as long as a conditional expectation function of that process satisfies a general regularity condition. In particular, within our framework, it is possible to consider the potential influence of the current charge level on the future charge accumulation which is neglected by the piece-wise linear assumption.

(2) In addition to the moments of total charge, it is derivable from our model of the joint distribution of the total charge and LOS, and the joint distribution of the costs and time being spent on every stage by every fixed time *t*. Therefore, our model provides more detailed information of the treatment that the patient experiences in hospital.

Although the motivation of our work is the analysis of the charge accumulation and the determination of hospital length of stay, it turns out that the proposed method is useful for many other problems where the relation among the time to event and a hidden continuous process as well as a jump process is in interest. For example, in the field of investment risk management, it is always important to detect how the default probability of the corporate bond issued by a firm is affected by the growth stage and profitability (say measured by the flow of revenue) of that firm. In this case, our model can definitely provide some insights if we identify the default as the event in interest and consider the revenue flow as determined by a continuous process similar to the charge accumulation and the transition among different growth stages of the firm as described by a CPH process. In addition to problems of the survival-type, it is also natural to extend our work to the case of competing risks, of which every stage in our model can be identified with a type of risk. Although in competing risk models, the CPH transition matrix is no longer sufficient, it turns out that the partition-based technique introduced below is extendible to derive the joint distributions of a wide class of the competing risk models, the details of which will be discussed in a related paper by the authors.

This paper is organized as follows. In Section 2, after a short review of the CPH models and RGRST models, we present the correspondence between them and briefly introduce the estimation algorithm. In Section 3, we conduct numerical studies to show the validity and usefulness of our model. A couple of interesting findings toward the medical outcome database, the New York State's Statewide Planning and Research Cooperative System 2013, are discussed.  Section 4 concludes the paper.

## 2. Model

In this section, a new model (denoted as CPH-RGRST model) is constructed that connects the CPH models to RGRST models in the sense that

(1) a CPH-RGRST model is a RGRST model;

(2) charges in a CPH-RGRST model can be classified into a number of stages such that every stage is identified with a phase in a given CPH model in the sense that, at every time *t*, the probability of staying in a stage *i* is exactly given by the probability in the *i*th phase of the CPH model.

In particular, the marginal distribution of LOS induced by a CPH-RGRST model is a CPH distribution. We shall state the detailed construction of the CPH-RGRST models after a brief review of the definition and some basic properties of RGRST models and CPH models.

### 2.1. the Joint Model (RGRST) versus the CPH Model

A RGRST model can be formally defined as follows as discussed in Gardiner et al. [18, 19] and Polverejan et al. [20]:

(1) $$\begin{array}{c} Y\left(\;t\;\right)=Y_{0}+\overset{t}{\underset{0}{\int }}I\left(\;T>s\;\right)\epsilon \left(\;s\;\right)ds, \end{array}$$

where the process {*Y*(*t*) : *t* ∈ [0, *∞*} represents the actual charge level at each time. The random variable *T* indicates the LOS, and *I* is the indicator function. {*I*(*T* > *t*) : *t* ∈ [0, *∞*} (*I*(*t*) for short) is the event process representing whether or not to stay in hospital for longer time at each time point *t*. {*ϵ*(*t*)} is a nonnegative process characterizing the potential increment rate of charge per unit time provided that patient decides to stay, and *Y*_0_ is a nonnegative random variable representing the charge at the initial time. We shall denote by *G*(*t*) = *Y*_0_ + ∫_0_^*t*^*ϵ*(*s*) *ds* the potential charge accumulation process in distinguishing the actual charge process {*Y*(*t*)}.

As shown in the supplementary materials (available here) note that a RGRST model can be completely specified by the initial probability density function (pdf), *p*(*y*, 0), induced by the initial charge *Y*_0_ and the following two conditional expectation functions:

(2) qy,t=Eϵt ∣ Gt=yρy,t=EIT>t ∣ Gt=y.

And using (2), the joint probability density function (pdf) of the LOS (*T*) and the total charge (*Y*_*T*_) at the discharge time *T* can be expressed as follows:

(3) $$\begin{array}{c} f\left(\;y,t\;\right)=p\left(\;y,t\;\right)\cdot \left(\;-\frac{\partial q}{\partial y}\cdot q-\frac{\partial q}{\partial t}\;\right)\left(\;y,t\;\right), \end{array}$$

where the function *p*(*y*, *t*) in variable *y* is the time-dependent pdf induced by *G*(*t*). The detailed derivation of (3) can be found in the supplementary materials. Expression (3) is useful in the estimation algorithm stated in the next section as it is the key component of the likelihood function.

To associate RGRST models with the CPH models, the hospital length of stay, represented as the random variable *T* in (1), should induce a CPH distribution generated from a CPH model, which is a finite-state continuous-time stationary Markovian process with only one absorbing state/phase (we shall use the term “phase”, by convention, in place of “state”). A CPH model is determined by an initial probability mass vector *α* with *α*_*i*_ ≥ 0 and ∑_*i*=1_^*n*^*α*_*i*_ = 1, and the transition intensity matrix

(4) $$\begin{array}{c} A=\left\{\begin{array}{cccccc} -c_{1}-\lambda_{1} & \lambda_{1} & 0 & \cdots & 0 & c_{1}\\ 0 & -c_{2}-\lambda_{2} & \lambda_{2} & \cdots & 0 & c_{2}\\ 0 & 0 & \ddots & \ddots & \vdots & \vdots \\ \vdots & \ddots & \ddots & \ddots & \lambda_{n-1} & c_{n-1}\\ 0 & 0 & \cdots & 0 & -c_{n} & c_{n}\\ 0 & 0 & \cdots & 0 & 0 & 0 \end{array}\right\}, \end{array}$$

where *λ*_*k*_, *c*_*k*_ > 0 and the entry *a*_*i*,*j*_ of *A* represents the transition intensity of a patient *ω* from phase *S*_*i*_ to phase *S*_*j*_ at every time *t* > 0; formally:

(5) $$\begin{array}{c} a_{i,j}=\underset{\delta ↓0}{\lim}\frac{Prob\left(\omega \in S_{j}\;\;at\;\;t+\delta \;|\;\omega \in S_{i}\;\;at\;\;t\right)}{\delta }. \end{array}$$

As suggested in McClean et al. [10], a phase in a CPH model can be identified with a treatment stage during hospital stay, such as diagnosis, acute care, assessment, rehabilitation, and long-stay care. The transition of patients among these stages characterizes the treatment progress.

### 2.2. Correspondence between CPH and RGRST Models

The main result of this section is that there does exist a correspondence between CPH and RGRST models. The correspondence is built through converting the continuous variable, charge, in a RGRST model to finite many discrete states by partitioning the product space, *ℝ*_+_ × *ℝ*_+_ (representing charge and time, respectively), into a number of cells such that each cell corresponds to a phase in a CPH model, while the evolution of the probability of staying in those cells is exactly determined by the given CPH model. More precisely, we have the following theorem.

**Theorem 1 .**

Fix a RGRST process {*Y*(*t*)} represented as a triple (*p*(*y*, 0), *q*, *ρ*) with *p*(*y*, 0) being the pdf of initial charge *Y*_0_ and *q*, *ρ* as defined in (2). Suppose functions *q*, *ρ*, and *p*(*y*, 0) are smooth and *q*, *ρ* satisfy

(6) $$\begin{array}{c} \frac{\partial \log\left(\rho \right)}{\partial y}\cdot q+\frac{\partial \log\left(\rho \right)}{\partial t}\equiv -c\;\rho >0 \end{array}$$

for some constant *c* > 0. Then, for any fixed positive integer *n*, an *n*-dimensional vector *α* > 0 with ∑_*i*=1_^*n*^*α*_*i*_ = 1, and an *n* − 1-dim vector *λ* > 0, there exists an *n*−partition of the space [0, *∞*^2^ denoted as *𝒫* such that the following time-dependent probability mass function *P*(*t*) defined on the *n* + 1 tuple {1,…, *n* + 1}:

(7) Pit=ProbYt∈Pi∩0,∞×t,IT>t=1,i∈1,…,nPn+1t=ProbIT>t=0,

is generated by a CPH model with the initial mass *α* and its transition matrix is given as in (4) with *c*_*i*_ ≡ *c* for *i* = 1,…, *n*.

The proof of Theorem 1 is presented in the Appendix. From the proof, it is clear that the connection between RGRST models and CPH models is equivalent to a constraint put on the conditional probability function *ρ* in (2) of the underlying RGRST model by the condition (6). In fact, the functional form of *ρ* is completely determined by (6) and the function *q*, which gives a first-order partial differential equation (PDE) of *ρ*. This equation turns out to be solvable and has a unique solution for a given boundary condition. Therefore, using the characteristic method, Evans [21], we can solve (6) and express the function *ρ* as follows:

(8) $$\begin{array}{c} \rho \left(\;y,t\;\right)=\rho_{b}\left(\;g\left(\;y,t,s^{*}\left(\;y,t\;\right)\;\right),s^{*}\left(\;y,t\;\right)\;\right)\cdot \exp\left(\;-c\cdot t\;\right), \end{array}$$

where *ρ* evaluated at *t* = 0 is constantly 1 which means that all patients have to stay in hospital for a positive time before discharge; the form of the boundary *b* and the value of the function *ρ* on *b* (denoted as *ρ*_*b*_) are constructed by iteratively solving the Initial Value Problem (IVP) (A.11) in the proof; the details of the iteration are presented in Algorithm 1 of Corollary 2. *s*^*∗*^ is the first time when the solution trajectory (*g*) of IVP (A.11) (starting from (*y*, *t*)) touches the boundary curve *b*. Equation (8) is crucial to determining the parametric form of the joint pdf (3) and the likelihood function used for estimation.

The next corollary is a direct result of Theorem 1. It extends the construction in Theorem 1 to a more general situation where the transition intensity from different transient states to the absorbing state does not have to be identical; i.e., *c*_*i*_ does not have to be equal to *c*_*j*_ for different *i*, *j*. Therefore, it is always possible to achieve an arbitrary CPH model from a RGRST model satisfying a generalized version of condition (6) with *c* replaced by *c*_*i*_ for different *i*.

**Corollary 2 .**

The smooth requirement on the function *ρ* in Theorem 1 can be replaced by the following weaker condition.

*Condition 3*. The function *ρ* is continuous and almost everywhere differentiable under the standard Lebesgue measure on [0, *∞*^2^ and has integrable partial derivatives.

*Under the Condition 3, for an arbitrary given CPH model represented by the transition matrix (4) and the initial probability mass vector α* = (*α*_1_,…, *α*_*n*_)*, there always exists a RGRST model together with a set of partition curves *{*C*_0_ ≡ 0 < *C*_1_ < ⋯<*C*_*n*_ ≡ *∞*}* such that the mapping*

(9) $$\begin{array}{c} \left\{\;\left(\;y,t\;\right):C_{i-1}\left(\;t\;\right)\leq y\leq C_{i}\left(\;t\;\right)\;\right\}\mapsto S_{i},\;i\in \left\{1,\ldots ,n\right\} \end{array}$$

*converts the RGRST model to the given CPH model, where S*_*i*_* is the ith phase in the CPH model.*

*Moreover, the desired RGRST model and the partition curves can be inductively constructed through Algorithm* 1 *, where “solve*(·)*” represents the operation to solve the equation “*·*”.*

Notice that given the partition curves {*C*_0_ ≡ 0 < *C*_1_ < ⋯<*C*_*n*−1_, *C*_*n*_ ≡ *∞*} and the joint pdf (3), deriving the conditional probability density of the cumulative charge *G*(*t*) is very simple given that at time *t* patients stay in the *i*th-stage, *S*_*i*_:

(10) ProbGt=y ∣ Si=pyi,t·ρyi,t∫Ci−1tCitpx,t·ρx,tdx·ICi−1t≤y≤Cit.

With the help of the conditional density (10), we can define a variety of measures of the “cost” of staying in a stage. For instance, fixing a stage and a time *t*, we can think of the price as the amount that has been charged since patients arrived in that stage for the first time, the daily price as the amount being charged per day within that stage, and the time cost as the length of time that patients have spent in that stage by *t*. Formally, the price (*Pr*_*i*_(*t*)), daily price (${\bar{Pr}}_{i}\left(t\right)$), and the time cost (*Ct*_*i*_(*t*)) for every stage and every time are defined as:

(11) Prit=∫Ci−1tCity−TYiy,t·py,t·ρy,tdy∫Ci−1tCitpy,t·ρy,tdyPr¯it=∫Ci−1tCity−TYiy,t/t−TTiy,t·py,t·ρy,tdy∫Ci−1tCitpy,t·ρy,tdyCtit=∫Ci−1tCitt−TTiy,t·py,t·ρy,tdy∫Ci−1tCitpy,t·ρy,tdy,

where *TT*_*i*_ is the conditional mean of the first arrival time into the stage *i* − 1 given the charge level *y* and current time *t*, and similarly, *TY*_*i*_ represents the conditional mean of the charge at the first arrival time to the stage *i* − 1 given *y* and *t*.

Although the price, daily price, and the time cost are defined through the first-order moment, the availability of the conditional probability (10) enables us to define the quantile version of (11). When there are large parts of outliers, a quantile version of those “cost” measures turns out to be more useful.

The information regarding the price and time spent in every such stage, as defined above, is helpful in rationalising the care process, thus reducing waste, in terms of unnecessary or inappropriate treatment, and avoiding delay, often the result of batch and queue processes, in a similar fashion to that adopted for industrial processes (McClean et al. [10]).

### 2.3. A Two-Stage Algorithm

Corollary 2 implies a two-step algorithm that uses the real hospital charge and LOS data as input to estimate the underlying CPH model and the RGRST model from which the CPH model is derived.

**Step 1 .**

Apply the full information maximum likelihood method (FML) and the marginal LOS data to estimate the transition matrix and the initial probability mass that determines the marginal CPH distribution of LOS. The resulting estimators are denoted as $\hat{\lambda }=\left({\hat{\lambda }}_{1},\ldots ,{\hat{\lambda }}_{n-1}\right)$, $\hat{c}=\left({\hat{c}}_{1},\ldots ,{\hat{c}}_{n}\right)$, and $\hat{\alpha }=\left({\hat{\alpha }}_{1},\ldots ,{\hat{\alpha }}_{n}\right)$.

**Step 2 .**

Apply Algorithm 1 to construct the function *ρ* from the estimators $\hat{\lambda }=\left({\hat{\lambda }}_{1},\ldots ,{\hat{\lambda }}_{n-1}\right)$, $\hat{c}=\left({\hat{c}}_{1},\ldots ,{\hat{c}}_{n}\right)$, and $\hat{\alpha }=\left({\hat{\alpha }}_{1},\ldots ,{\hat{\alpha }}_{n}\right)$, and construct the joint pdf of charge and LOS by formula (3). With the joint pdf, construct the likelihood function and apply FML to estimate the remaining parameters, which are used to characterize the function *q* and the initial density *p*(·, 0) (denoted by $\hat{params}$).

The use of FML guarantees that all estimators obtained from the two-stage algorithm are consistent and asymptotically normal-distributed.

## 3. Numerical Studies

In this section, we conduct the numerical studies to show the validity of our two-stage estimation procedure. We will apply our procedure to both of the real data and simulation sample.

Our data source is the medical outcome database, New York State's Statewide Planning and Research Cooperative System 2013 (SPARCS 2013). The histogram of the entire SPARCS 2013 indicates that the total charge approximately follows a log-normal distribution; therefore, we will take the following parametric form for the function *q*:

(12) $$\begin{array}{c} q\left(\;y,t\;\right)=y, \end{array}$$

and the initial *Y*_0_ is assumed to satisfy

(13) $$\begin{array}{c} \log Y_{0}\sim N\left(\;\mu ,\sigma \;\right). \end{array}$$

It turns out that under (12), (13), and (6), the resulting marginal distribution of total charge is close to a log-normal distribution.

When covariates exist (denoted by *X*), we assume that the random vector (*ε*_*Y*_, *ε*_*T*_) is independent from the covariate vector, and (exp⁡(*ε*_*Y*_), exp⁡(*ε*_*T*_)) follows the joint pdf given by (3) with the initial pdf, *q*, and *ρ* specified as in (13), (12), and (6), respectively. The covariates are linked with the total charge, *Y*_*T*_, and LOS, *T*, through the following regression equations:

(14) log⁡YT=θ0+θ·X+εYlog⁡T=β0+β·X+εT.

*θ* ^+^ = (*θ*_0_; *θ*), *β*^+^ = (*β*_0_; *β*) are the regression coefficient vectors.

As for the dimension of the underlying CPH model, we follow the convention in the previous studies of Faddy et al. [5]; Tang [4]; McClean et al. [10] and only consider the two cases where the number of nonabsorbing phases is 3 and 4. After a preliminary study, we select the 4-Phase CPH model as it can generate better fitting to the SPARCS data.

Under the specification above, there are three classes of parameters to estimate. They are (1) the parameter vectors *α*, *c* and *λ* involved in the CPH model, (2) the parameter (*μ*, *σ*) involved in the initial pdf, and (3) the regression coefficients (*θ*^+^, *β*^+^). We call the parameters of types (1) and (2) as the dynamic parameters because they specify the joint model that generates the distribution of charge and LOS.

In the real data study, we draw 5000 subsamples from SPARCS 2013 with the covariates consisting of the Severity of Illness, Mortality Risk of Illness (In SPARCS 2013, both of the two variables, Severity and Mortality, are quantified through a grading score, which is a number in the set {1,2, 3,4}.), and 24 categorical variables which represent 25 Major Diagnosis Codes (MDC), each of which associates with a class of illness. The summary statistics of our subsample verses the entire SPARCS 2013 with respect to the covariates are described in Table 1.

In the simulation study, we generate 5000 samples from a joint model without covariates and the true value of the dynamic parameters is taken as the estimated value from the real data study, which is given as in Table 2.

In both of the real data and simulation studies, the computer code is written in the language of Python 2.7 with python-scipy, python-numpy libraries being used.

### 3.1. Simulation Study

**Figure 1:**
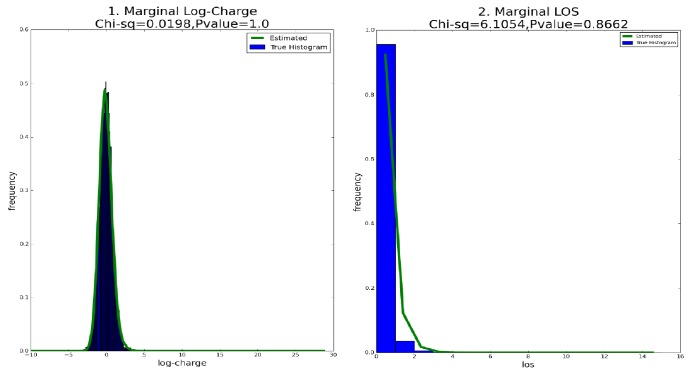
Goodness of Fit. Plots 1 and 2 are the fitted marginal CPH-RGRST distribution versus empirical histogram for log-charge and LOS.

The goodness of fit is measured through comparing the fitted curves and the empirical histogram (drawn from the simulation sample) for both of the marginal charge and LOS, as shown in first line of Figure 1. We conduct Pearson's *χ*^2^ test; the value of the *χ*^2^ statistics and the associated *P* values are (0.0171, 1.0) for the marginal charge and (6.1054, 0.8662) for the marginal LOS. From both of the fitting plots and the results of *χ*^2^ test, our fitting is fairly good.

We also evaluate the goodness of fit in terms of the joint distribution through Pearson's *χ*^2^ test; the *χ*^2^ statistics and its *P* value are (0.1911, 1.0), which is consistent with Figure 1. Therefore, the simulation study verifies the effectiveness of our two-step estimation procedure.

### 3.2. Real Example Study

#### 3.2.1. Regression Coefficients

The estimated regression coefficients are reported in Table 3, from which both of the severity and mortality of illness have significantly positive effect on both of the total charge and LOS that is consistent with the intuition.

On the other hand, among all the MDC groups, the Newborn And Other Neonates (MDC_15) and the Diseases and Disorders of the Musculoskeletal System And Connective Tissue (MDC_8) has the greatest negative and positive effects on the total charge, respectively, which is also consistent with the intuition. In contrast, the MDC groups with greatest negative and positive effect on LOS are the Diseases and Disorders of the Ear, Nose, Mouth and Throat (MDC_3) and the Mental Diseases and Disorders (MDC_19), respectively.

In addition, it turns out that the effects of different illnesses on the charge and LOS are not always homogeneous. There are a couple of MDC groups which affect the total charge and LOS in distinct direction. They are the Diseases and Disorders of the Nervous System (MDC_1), Diseases and Disorders of the Circulatory System (MDC_5), Diseases and Disorders of the Male Reproductive System (MDC_12), Diseases and Disorders of the Female Reproductive System (MDC_13), and Alcohol/Drug Use or Induced Mental Disorders (MDC_20). Among them, except the MDC_20 group, all the other groups have a more expensive bill but shorter hospital stay, and therefore a higher daily charge. In contrast, patients with alcohol/drug abuse tend to pay less but stay in hospital longer.

Combining the estimated coefficients in Table 3 and dynamic parameter in Table 2, we can even identify the stage from which a patient exits to discharge. The discharge stage encodes critical information of the treatment pathways and is important for management purpose.  Table 4 reports the estimated conditional mean (log-)charge, LOS, severity, and mortality risk for patients who exit from every stage. It is clear that the value of all the four variables monotonically increases as patients get discharged from later stages. Especially for severity and LOS, there are clear jumps from stages 1 and 2 to stages 3 and 4. The mean severity is doubled when transiting from stage 2 to stage 3 while the mean LOS almost gets tripled. This gap suggests that patients who are diagnosed with more severe conditions during admission are more likely to go over the treatment in stage 3 or 4, while their rehabilitation usually takes more time and more medical resources (McClean et al. [10]). This observation is consistent with the intuition and, to some extent, verifies the viewpoint that interprets the entire treatment process as a series of transitions among multiple medical stages.

#### 3.2.2. Cost

As discussed in the end of Section 2.1, the CPH-RGRST model enables us to evaluate the “cost” of each medical stage in different manners. Using the estimation results provided in the previous section, we can numerically compute the “cost” for our SPARCS sample.

**Figure 2:**
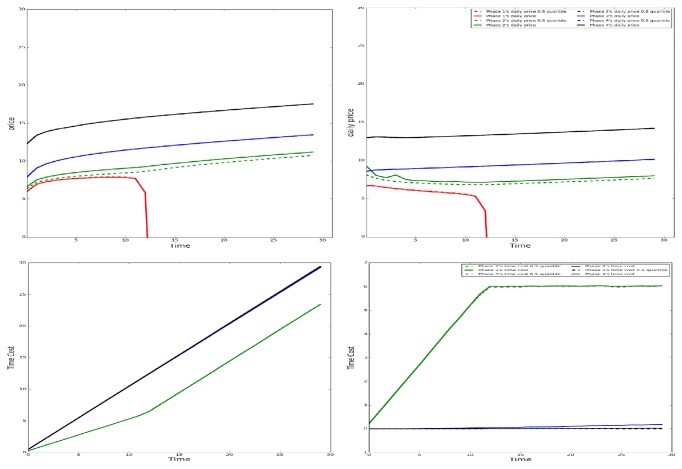
Cost of Stages. Plots 1, 2, and 3 sketch the log of the price, daily price, and the time cost (as defined in (11)) versus their quantile version, respectively. Plot 4 shows the other version of the time cost ( = *s* − *Ct*_*i*_(*s*) with *s* > 0) versus its quantile version.

**Figure 3:**
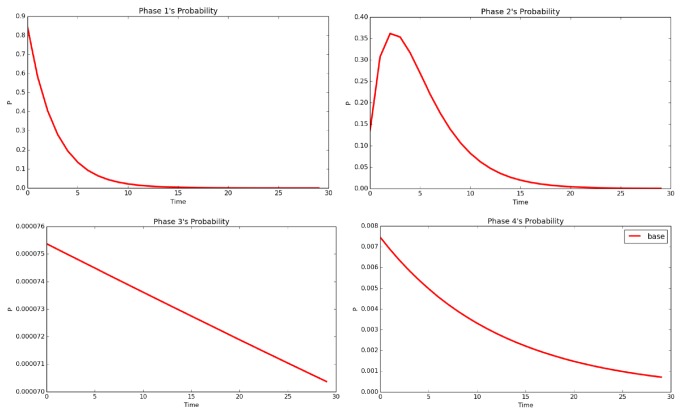
Probability of staying in every stage by time.

In Figure 2, we plot the estimated mean price, mean daily price, and mean time of staying for each of the four stages of the CPH model, where the “mean” refers to the CPH-RGRST process that generates the mean charge and LOS, ${\bar{Y}}_{T}=\exp\left(\varepsilon_{Y}\right)\exp\left(E_{X}\left(\hat{\theta_{0}}+\hat{\theta }X\right)\right)$, and $\bar{T}=\exp\left(\varepsilon_{T}\right)\exp\left(E_{X}\left(\hat{\beta_{0}}+\hat{\beta }X\right)\right)$. In Figure 3, the probability of staying in every nonabsorbing stage is plotted against the time. There are the following three major findings.

(i) From the plot 2 in Figure 2, the daily price for stage 1 declines over time, which is caused by the fact that, for those long-stay patients, they must have already switched into the higher stage treatments after the preexam period (represented by stage 1), which is very well captured in plot 1 of Figure 3. In contrast, for all the stage 2, 3, and 4, the daily price inclines to grow up in long run, which rejects the piece-wise linear assumption claimed in McClean et al. [11]. In fact, in contrast to the constant growth rate of charge within each stage, the increasing growth rate tends to be more reasonable, because a longer stay usually implies a worse health condition for a patient, who, therefore, needs better care, including more expensive medicines, more frequent exams, and the like. These items lift up the cost of stay per day. The same reasoning also applies well to the observation that the time cost of all stages is increasing over time as shown in plots 3 and 4 in Figure 2.

(ii) Although the time cost is slightly lower in stage 3 than in stage 4, both of the two stages (by (11), the time cost for stage 1 is trivial and constantly equal to the total time in hospital, so we omitted it in Figure 2) have their time cost almost identical to the total time that patients spent in hospital since they were admitted. In contrast, the time cost of stage 2 displays quite different features, which is not only much lower than that of the other stages, but, within the first 13 days, its growth rate is also slower. The different features of stage 2 are consistent with the estimated dynamic parameters in Table 2 and plot 2 in Figure 3. From Table 2, it is clear that the intensity of switch-in and switch-to-discharge in stage 2 is significantly higher than in the other stages, which means that there are two factors that lower down the time cost at stage 2. (1) There are a large portion of patients switching from stage 1 to stage 2 in the early time (<5 days, see plots 1 and 2 in Figure 3); in contrast there is almost no patient who could switch from lower stage to stage 3 or 4 (see plots 3 and 4 in Figure 3), which implies on average that the first arrival time to stage 2 is later than to stages 3 and 4. (2) The portion of patients switching out of stage 2 (mainly to discharge by plots 3 and 4 in Figure 3) is also high, which is not the case for stages 3 and 4 (reflected as the scale of plots 3 and 4 being much smaller than plots 1 and 2 of Figure 3). Therefore, the switch-out time from stage 2 is earlier than from stage 3 or 4 on average.

Factors (1) and (2) shown in Figure 2 and Table 2 indicate that stage 2 should associate to the major treatment procedures, like the main surgery, that most inpatients have to experience when staying in hospital. In fact, it is usual that patients need a couple of days as the preparation period before the main surgery, such as the period for preexams. This preparation period is exactly captured by factor (1) of stage 2. On the other hand, patients usually recovered soon after the main treatment procedure gets done, and then are discharged, which is reflected by factor (2) of stage 2.

(iii) The third interesting observation from Figure 2 is regarding the median price of stage 2. From plots 1 and 2 in Figure 2, we can see that only at stage 2 there is a clear deviation between the expectation version and the median version of the price. More precisely, at stage 2 the median price is significantly lower than the price defined through the first-order moment, and this fact holds at almost all the time *t* and also holds for the daily price. It is well known that when the median of a distribution is below its first-order moment, there exists a group of outliers with extremely great value. In the other words, Figure 2 indicates that a portion of patients in stage 2 are charged much higher than the others in that stage and all the time. From the perspective of patient's welfare and the effective allocation of medical resources, it is meaningful to have some further researches in identifying the causes that make some patients in stage 2 being charged more.

**Remark 3 .**

By Figure 3, it is clear that the probability of staying in stages 3 and 4 is small over all time. This fact might be induced by model overfitting as pointed out by a referee, but it is not. In contrast, the low probability reflects a deep-level distributional property of SPARCS data. A very large proportion of inpatients recorded in SPARCS only have extremely short hospital stay and 99+ percent of them get discharged by day 10, while no more than 0.01 percent of patients can stay in hospital for more than 25 days. But at the meantime, there do exist a small group of patients who can live in hospital for a couple of months before discharge. The same pattern can be observed for the total charge; the most expensive expenditure can take million dollars while more than 99 percent of patients are charged no more than 100,000 dollars.

Based on the observation above and the nondecreasing design of the CPH-RGRST models that higher stages correspond to longer hospital stay and higher total costs, the low probability of staying in the highest two stages just reflects a fact that both of the charge and LOS data in SPARCS 2013 have a very long and thin tail to the right. This tail property may not be well fitted if a CPH-RGRST model with fewer phases is used, because there will not be enough freedom to distinguish the portion of patients in the tail from those whose charge and LOS stay around the mode.

## 4. Summary

We introduced a methodology whereby the widely used CPH models and RGRST models can be combined together and a variety of measures of the cost of phases in the CPH model can be defined. A two-step procedure is proposed to estimate the combined CPH-RGRST model and the simulation study is done to verify the effectiveness of the estimation procedure. With the data sampled from SPARCS 2013, we estimated a four-phase CPH-RGRST model and drew the cost curves for every phase. To distinguish the effect of different types of illness on the charge and LOS distribution, we incorporated MDC groups and the severity and mortality risk of illness as covariates into the estimation.

We found that the effect of illness on the total charge and LOS is not always homogeneous. In particular, there are five MDC groups that affect the charge and LOS in different direction. Among them, there is only one MDC group, representing the alcohol/drug abuse, which has the negative effect on the final charge while it lifts up the LOS drastically.

The daily charge for all the stages, 2, 3, and 4, is increasing over time. This fact implies a nonlinear charge accumulation process within every stage and therefore contradicts the piece-wise linear assumption used by the other authors, McClean et al. [11]. We believe that the increasing daily charge is more realistic and reflects dynamic interaction between the health condition of patients and the treatment they accept.

Among all the four stages, stage 2 shows quite different features in both the price measure and the time cost measure. In terms of the time cost, stage 2 is significantly lower than stages 3 and 4 almost all the time. This observation is consistent with the relatively high switch-in and switch-to-discharge intensity that the stage 2 has and associates the stage 2 with the major treatment procedures that most patients need to experience when staying in hospital.

The median is much lower than the mean of both the price and daily price in stage 2, while this kind of deviation does not exist for the other stages, and it implies that there is a portion of outlier patients who are charged much more than the other patients in stage 2. We believe that further studies are needed to find out the causes of those patients being charged more in stage 2, since it matters to the efficiency of the allocation of medical resources.

## Figures and Tables

**Algorithm 1:**
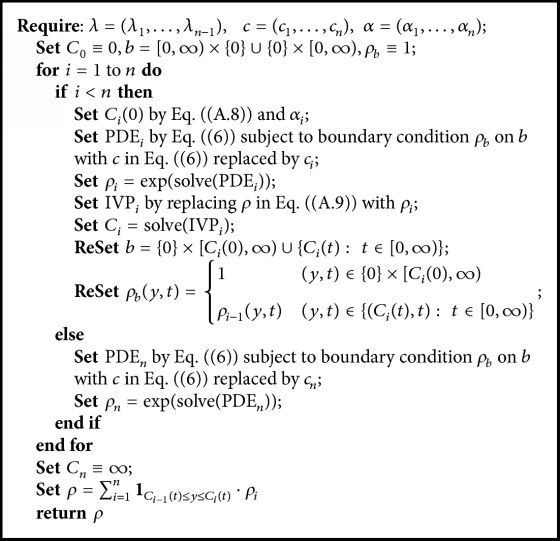
Construct_*ρ*.

**Table 1:** Descriptive statistics of SPARCS 2013.

Characteristics	Group	N (%)	Sample_N (%)	LOS (SD)	Sample_LOS (SD)	Charge (SD)	Sample_Charge (SD)
All Patients		2418874 (100)	5000 (100)	5.46 (8.11)	5.51 (8.16)	36931.77 (68973.47)	36861.8 (67053.64)

MDC	0.0	17.0 (0.0)		11.0 (24.69)		102910.82 (280754.64)	
	1.0	142651.0 (5.9)	298.0 (5.96)	5.7 (8.69)	5.01 (6.14)	46962.08 (83724.59)	41911.53 (50501.65)
	2.0	4138.0 (0.17)	13.0 (0.26)	3.62 (5.18)	3.38 (1.89)	27185.04 (37576.18)	28478.72 (22611.85)
	3.0	32743.0 (1.35)	72.0 (1.44)	3.59 (5.59)	2.81 (2.72)	29468.92 (50592.3)	22093.15 (20516.67)
	4.0	206374.0 (8.53)	425.0 (8.5)	5.81 (7.64)	5.42 (6.99)	37165.6 (64478.26)	35254.34 (48416.02)
	5.0	320765.0 (13.26)	655.0 (13.1)	4.78 (6.58)	4.68 (5.4)	50065.14 (84839.8)	48514.49 (67896.89)
	6.0	211325.0 (8.74)	461.0 (9.22)	5.11 (6.63)	5.56 (7.16)	35785.32 (54820.39)	37176.32 (45615.65)
	7.0	65928.0 (2.73)	116.0 (2.32)	5.6 (6.96)	4.91 (4.31)	42718.49 (78816.64)	34176.78 (38341.19)
	8.0	201134.0 (8.32)	419.0 (8.38)	4.91 (5.95)	5.0 (5.35)	50655.45 (55819.15)	50609.01 (45334.44)
	9.0	66120.0 (2.73)	136.0 (2.72)	4.6 (5.95)	5.07 (8.42)	28073.74 (37308.49)	28829.12 (29869.57)
	10.0	74993.0 (3.1)	171.0 (3.42)	3.97 (5.83)	4.05 (4.72)	28568.47 (43837.41)	27236.6 (30456.03)
	11.0	103597.0 (4.28)	221.0 (4.42)	5.43 (6.75)	5.09 (5.1)	36812.91 (53368.81)	33884.47 (38131.54)
	12.0	11181.0 (0.46)	21.0 (0.42)	3.44 (6.27)	4.81 (10.56)	30593.31 (30945.72)	39233.29 (46533.28)
	13.0	31682.0 (1.31)	57.0 (1.14)	3.13 (5.23)	2.47 (2.03)	28998.31 (33592.18)	31389.63 (20325.52)
	14.0	257203.0 (10.63)	504.0 (10.08)	2.91 (2.54)	2.88 (2.47)	16435.92 (17226.17)	16714.7 (18104.7)
	15.0	236599.0 (9.78)	439.0 (8.78)	3.78 (7.99)	4.06 (7.8)	17912.83 (85865.5)	18682.72 (72830.49)
	16.0	37899.0 (1.57)	92.0 (1.84)	5.01 (6.87)	4.77 (3.79)	37100.38 (83604.25)	36537.56 (52336.47)
	17.0	22289.0 (0.92)	55.0 (1.1)	9.57 (12.73)	9.38 (11.59)	87130.44 (139632.35)	81519.0 (128268.96)
	18.0	108416.0 (4.48)	224.0 (4.48)	9.09 (10.7)	10.24 (15.07)	63423.77 (99592.33)	80804.11 (200106.77)
	19.0	116683.0 (4.82)	245.0 (4.9)	12.94 (16.11)	12.62 (17.7)	34162.28 (57058.45)	32507.77 (49653.33)
	20.0	75432.0 (3.12)	170.0 (3.4)	6.34 (7.45)	6.6 (7.65)	17400.15 (23797.61)	17228.44 (20575.0)
	21.0	30203.0 (1.25)	71.0 (1.42)	4.29 (7.18)	4.77 (10.44)	31248.52 (64435.64)	33845.59 (75320.46)
	22.0	1929.0 (0.08)	2.0 (0.04)	9.06 (13.5)	8.0 (2.83)	79337.2 (184652.6)	51080.31 (29187.33)
	23.0	46924.0 (1.94)	106.0 (2.12)	10.87 (10.27)	11.18 (8.88)	46721.27 (52350.27)	45356.56 (37999.3)
	24.0	8733.0 (0.36)	20.0 (0.4)	8.6 (11.36)	8.55 (8.81)	57383.57 (105543.15)	40839.06 (39649.57)
	25.0	3916.0 (0.16)	7.0 (0.14)	10.77 (12.01)	11.0 (7.44)	103841.73 (118285.21)	73790.14 (54114.8)

Severity	0.0	40.0 (0.0)		6.35 (16.4)		47710.78 (186214.68)	
	1.0	881300.0 (36.43)	1760.0 (35.2)	3.09 (3.97)	3.02 (3.37)	20164.74 (25917.49)	20249.04 (23995.3)
	2.0	929347.0 (38.42)	1939.0 (38.78)	4.96 (6.89)	5.16 (7.76)	30512.25 (37884.57)	30602.25 (38507.07)
	3.0	479712.0 (19.83)	1048.0 (20.96)	7.73 (8.46)	7.57 (7.68)	51935.05 (65352.31)	51307.28 (61645.27)
	4.0	128475.0 (5.31)	253.0 (5.06)	16.83 (18.2)	17.06 (18.36)	142361.38 (210806.88)	140564.83 (210012.56)

Mortality	Extreme	106154.0 (4.39)	210 (4.2)	14.96 (16.66)	13.81 (15.11)	129939.83 (200746.65)	114408.34 (172257.11)
	Major	311482.0 (12.88)	692 (13.84)	8.69 (10.14)	8.51 (9.96)	61247.22 (92604.92)	64073.56 (108815.34)
	Minor	1482115.0 (61.27)	3007 (60.14)	4.03 (6.16)	4.02 (6.31)	24133.09 (33016.21)	23905.06 (31377.21)
	Moderate	519083.0 (21.46)	1091 (21.82)	5.67 (7.03)	6.12 (7.96)	39863.27 (55375.06)	40386.64 (51083.0)

**Table 2:** Estimated dynamic parameters.

Dynamic Parameters	Values
(*μ*, *σ*)	(−0.5715, 0.7149)
*α*	(0.9922, ≈0.0, 0.0001, 0.0077)
*c*	(≈0.0, 4.4796, ≈0.0, 0.9934)
*λ*	(4.4905, ≈0.0, 0.029)

**Table 3:** Estimated regression coefficients.

Groups	Log-Charge (*P* values)	Log-LOS (*P* values)
Intercept	9.3245 (<0.0001)	1.7512 (<0.0001)
MDC_1	0.0697 (<0.0001)	−0.1008 (<0.0001)
MDC_2	0.2756 (<0.0001)	0.519 (<0.0001)
MDC_3	−0.1522 (<0.0001)	−0.3564 (<0.0001)
MDC_4	−0.2062 (<0.0001)	−0.187 (<0.0001)
MDC_5	0.1018 (<0.0001)	−0.2451 (<0.0001)
MDC_6	−0.0072 (<0.0001)	−0.1214 (<0.0001)
MDC_7	0.0686 (<0.0001)	−0.0432 (<0.0001)
MDC_8	0.5344 (<0.0001)	0.0258 (<0.0001)
MDC_9	−0.1364 (<0.0001)	−0.1597 (<0.0001)
MDC_10	−0.1132 (<0.0001)	−0.2823 (<0.0001)
MDC_11	−0.265 (<0.0001)	−0.2736 (<0.0001)
MDC_12	0.1164 (<0.0001)	−0.3405 (<0.0001)
MDC_13	0.0504 (<0.0001)	−0.2928 (<0.0001)
MDC_14	−0.3242 (<0.0001)	−0.1945 (<0.0001)
MDC_15	−1.0351 (<0.0001)	−0.0311 (<0.0001)
MDC_16	−0.0948 (<0.0001)	−0.1035 (<0.0001)
MDC_17	0.2244 (<0.0001)	0.1522 (<0.0001)
MDC_18	−0.0289 (<0.0001)	−0.06 (<0.0001)
MDC_19	0.0381 (<0.0001)	0.9866 (<0.0001)
MDC_20	−0.5335 (<0.0001)	0.2749 (<0.0001)
MDC_21	−0.2574 (<0.0001)	−0.1887 (<0.0001)
MDC_22	0.3689 (<0.0001)	0.3077 (<0.0001)
MDC_23	0.1332 (<0.0001)	0.51 (<0.0001)
MDC_24	−0.2957 (<0.0001)	−0.2083 (<0.0001)
APR Risk of Mortality	0.1436 (<0.0001)	0.18 (<0.0001)
APR Severity of Illness	0.3605 (<0.0001)	0.3338 (<0.0001)

**Table 4:** Summary of discharge stages.

	Stage 1	Stage 2	Stage 3	Stage 4
severity	0.099	0.454	1	2.238
mortality	1.524	1.814	3	3.355
charge	8.062	9.842	11.525	11.505
LOS	2.48	5.732	13	14.87

## Data Availability

The data used in this paper is sampled from New York State's Statewide Planning and Research Cooperative System 2013, which is publicly available online at the following url: https://health.data.ny.gov/Health/Hospital-Inpatient-Discharges-SPARCS-De-Identified/npsr-cm47. The specific sample used in this study and the python code for processing the data and implementing the estimation and simulation study are available from the corresponding author on request.

## Appendix

### Proof of Theorem 1

The main idea of the proof is to construct the partition *𝒫* by induction. Assume, firstly, that the partition *𝒫* is formed by *n* curves in [0, *∞* (denoted as (*t*, *C*_*i*_(*t*)) for *i* ∈ {0,1,…, *n* − 1}) satisfying increasing condition as follows:

(A.1) Ci+1t>Cit≥0C0t≡0

in such a way that *𝒫*_*i*_ = {(*y*, *t*) : *C*_*i*−1_(*t*) ≤ *y* < *C*_*i*_(*t*)} for *i* ∈ {1,…, *n*} (for simplicity of notation, we assume *C*_*n*_(*t*) : ≡*∞*.). So, to prove the theorem, it suffices to find out a family of increasing curves {*C*_*i*_ : *i* ∈ {0,1,…, *n* − 1}} with the induced transition matrix being as stated in the theorem.

Firstly, notice that, by condition (6), we have for *i* ∈ {0,1,…, *n* − 1} and any family of n curves {*C*_*i*_ : *i* ∈ {0,1,…, *n* − 1}} satisfying increasing condition (A.1) the fact that the following holds:

(A.2) limδ↓0⁡ProbIT>t+δ=0 ∣ Yt∈Cit,Ci+1t,IT>t=1δ=∫CitCi+1t−∂ρ/∂y·q+∂ρ/∂ty,t·py,tdy∫CitCi+1tρy,t·py,tdy≡c.

Moreover, because

(A.3) $$\begin{array}{c} Prob\left(\;Y\left(\;t\;\right)\geq C_{n}\left(\;t\;\right)=+\infty ,I\left(\;T>t\;\right)=1\;\right)=0, \end{array}$$

we can conclude that no matter the choice of {*C*_*i*_ : *i* ∈ {0,1,…, *n* − 1}}, the transition probability matrix *A* always has its column *n* + 1 of the following form:

(A.4) An+1=c⋮c0

On the other hand, the increasing condition (A.1) and the nonincreasing property of the event processes *I*(*t*) guarantee that for all *i*, *j* ∈ {0,1,…, *n* − 1} with *j* ≠ *i* or *i* + 1 and small enough *δ*

(A.5) ProbYt+δ∈Cjt,Cj+1t ∣ Yt∈Cit,Ci+1t,IT>t=1=0,ProbYt+δ∈Cjt,Cj+1t,IT>t+δ=1 ∣ IT>t=0=0.

That is, the induced transition matrix *A* satisfies

(A.6) Ai,j=0,j∉i,i+1,  i,j∈1,…,n,An+1,j=0,j∈1,…,n

So, to prove the theorem, it suffices to construct a family of increasing curves {*C*_*i*_ : *i* ∈ {0,1,…, *n* − 1}} guaranteeing that

(A.7) Ai,i+1=λi,i∈1,…,n−1,Pi0=αi,i∈1,…,n−1

However, these two conditions are equivalent to finding

(1) a sequence {*c*_*i*_ : *i* ∈ {0,1,…, *n*}} with *c*_0_ = 0 and *c*_*n*_ = *∞* such that

(A.8) $$\begin{array}{c} \alpha_{i}=\overset{c_{i}}{\underset{c_{i-1}}{\int }}p\left(\;y,0\;\right)dy,\;i\in \left\{1,\ldots ,n-1\right\}; \end{array}$$

(2) a sequence of solutions {*y*_*i*_(*t*) : *i* ∈ {0,1,…, *n* − 1}} with *y*_0_ ≡ 0 to initial value problems (IVP) for *i* ∈ {1,…, *n* − 1}

(A.9) dyidt=qyi,t−λi·∫yi−1yipx,t·ρx,tdxpyi,t·ρyi,tyi0=ci

Condition (A.9) arises from the following equality:

(A.10) limδ↓0⁡ProbYt+δ∈Cit+δ,Ci+1t+δ,IT>t+δ=1 ∣ Yt∈Ci−1t,Cit,IT>t=1δ=limδ↓0⁡ProbYt+δ∈Cit+δ,Ci+1t+δ,IT>t+δ=1,Yt∈Ci−1t,Cit,IT>t=1δ·ProbYt∈Ci−1t,Cit,IT>t=1=limδ↓0⁡∫Cit+δg−1Cit,t,t+δpy,t+δ·ρy,t+δdyδ·∫Ci−1tCitpy,t·ρy,tdy=pCit,t·ρCit,t·limδ↓0⁡g−1Cit,t,t+δ−g−1Cit,t,t/δ−dCit/dt∫Ci−1tCitpy,t·ρy,tdy=pCit,t·ρCit,t·qCit,t−dCit/dt∫Ci−1tCitpy,t·ρy,tdy=λi,

where the time-dependent density function *p* of the potential charge process {*G*(*t*)} has the form of *p*(*y*, *t*) = *p*(*g*(*y*, *t*, *t*), 0) · ∂*g*(*y*, *t*, *t*)/∂*y*. The involved function *g*, viewed as a family of functions in variable *s*, solves the following family of IVPs:

(A.11) dydt=−qy,t−sgy,t,0=y

and *g*^−1^ is defined as the inverse to *g* such that *g*^−1^(*y*, 0, *t*) = {*x* : *g*(*x*, *t*, *t*) = *y*}.

It is easy to check that condition (1) does always hold. Finally, thanks to the existence and uniqueness theorem of solutions to IVPs, the solution curves

(A.12) $$\begin{array}{c} \left\{\;y_{i}\left(\;t\;\right):i\in \left\{\;0,1,\ldots ,n-1\;\right\}\;\right\} \end{array}$$

do exist to the IVPs in (A.9).

## Supplementary Materials

Supplementary Material A note on the joint probability density function of a stochastic growth process with random stopping time. The technical proof of expression (3) is too complicated and left in the supplemental note.
