# Supplementary material for: Understanding Dynamic Status Change of Hospital Stay and Cost Accumulation via Combining Continuous and Finitely Jumped Processes
Source: Comput Math Methods Med. 2018 Jun 10;2018:6367243. doi: 10.1155/2018/6367243 (PMC6015722; doi:10.1155/2018/6367243)
Supplement: Supplementary Material — A note on the joint probability density function of a stochastic growth process with random stopping time. The technical proof of expression (3) is too complicated and left in the supplemental note. [file 6367243.f1.docx]

A note on the joint probability density function of a stochastic growth process with random stopping time. The technical proof of the expression (2.3) is too much complicated and left into the supplemental note.
